# Supplementary material for: Haplotype-resolved genome assembly enables gene discovery in the red palm weevil Rhynchophorus ferrugineus
Source: Sci Rep. 2021 May 11;11:9987. doi: 10.1038/s41598-021-89091-w (PMC8113489; doi:10.1038/s41598-021-89091-w)
Supplement: Supplementary file 1 — Supplementary Information 1 [file 41598_2021_89091_MOESM1_ESM.pdf]

Supplementary material for:

Haplotype-resolved genome assembly enables gene discovery in the red palm weevil *Rhynchophorus ferrugineus*

Guilherme B. Dias[1,*], Musaad A. Altammami[2], Hamadttu A.F. El-Shafie[3], Fahad M. Alhoshani[2], Mohamed B. Al-Fageeh[2], Casey M. Bergman[1], Manee M. Manee[2,*]

[1] Department of Genetics and Institute of Bioinformatics, University of Georgia, Athens, GA 30602, USA [2] National Centre for Biotechnology, King Abdulaziz City for Science and Technology, Riyadh 11442, Saudi Arabia [3] Date Palm Research Center of Excellence, King Faisal University, Al-Ahsa 31982, Saudi Arabia

[*]Corresponding authors: guilherme.dias@uga.edu, malmanee@kacst.edu.sa

Supplementary Table S1: **Species and genome assemblies used for protein sequence evidence in the BRAKER annotation of RPW pseudo-haplotype1.**

Species	Order	Family	Accession
Aethina tumida	Coleoptera	Nitidulidae	GCF_001937115.1
Agrilus planipennis	Coleoptera	Buprestidae	GCF_000390285.2
Anoplophora glabripennis	Coleoptera	Cerambycidae	GCF_000699045.2
Asbolus verrucosus	Coleoptera	Tenebrionidae	GCA_004193795.1
Callosobruchus maculatus	Coleoptera	Chrysomelidae	GCA_900659725.1
Drosophila melanogaster	Diptera	Drosophilidae	GCF_000001215.4
Dendroctonus ponderosae (female)	Coleoptera	Curculionidae	GCA_000346045.2
Dendroctonus ponderosae (male)	Coleoptera	Curculionidae	GCF_000355655.1
Diabrotica virgifera	Coleoptera	Chrysomelidae	GCF_003013835.1
Ignelater luminosus	Coleoptera	Elateridae	GCA_011009095.1
Leptinotarsa decemlineata	Coleoptera	Chrysomelidae	GCF_000500325.1
Nicrophorus vespilloides	Coleoptera	Silphidae	GCF_001412225.1
Onthophagus taurus	Coleoptera	Scarabaeidae	GCF_000648695.1
Oryctes borbonicus	Coleoptera	Scarabaeidae	GCA_001443705.1
Photinus pyralis	Coleoptera	Lampyridae	GCF_008802855.1
Sitophilus oryzae	Coleoptera	Curculionidae	GCF_002938485.1
Tribolium castaneum	Coleoptera	Tenebrionidae	GCF_000002335.3

Supplementary Table S2: **Summary of nucleotide differences between RPW pseudo-haplotype1 and pseudo-haplotype2 assemblies.**

	$\geq$1 kb		$\geq$10 kb		$\geq$50 kb	
Variant type	# differences	differences / kb	# differences	differences / kb	# differences	differences / kb
Substitution	2,056,517	3.5837	1,950,309	3.9107	1,078,482	3.0011
1 bp deletion	48,050	0.0837	45,656	0.0915	25,720	0.0716
1 bp insertion	47,731	0.0832	45,382	0.0910	25,550	0.0711
2 bp deletion	16,522	0.0288	15,751	0.0316	8,917	0.0248
2 bp insertion	16,286	0.0284	15,520	0.0311	8,776	0.0244
3-50 bp deletion	61,144	0.1066	57,941	0.1162	32,540	0.0906
3-50 bp insertion	61,557	0.1073	58,399	0.1171	32,694	0.0910
50-1000 bp deletion	9,864	0.0172	9,363	0.0188	5,233	0.0146
50-1000 bp insertion	9,519	0.0166	9,057	0.0182	5,007	0.0139
1 kb+ insertion	375	0.0007	370	0.0007	211	0.0006
1 kb+ deletion	358	0.0006	347	0.0007	195	0.0005

Numbers of absolute differences and numbers of differences per 1 kb are shown for orthologous minimap2 alignments longer than 1 kb, 10 kb and 50 kb, respectively. Inclusion of shorter alignments increases the absolute number of differences identified, but the corresponding increase in genomic regions covered leads to only minor changes in normalized levels of differences between pseudo-haplotypes. The total aligned sequence length is 573,852,952 bp for alignments $\geq$1 kb, 498,705,929 bp for alignments $\geq$10 kb, and 359,359,388 bp for alignments $\geq$50 kb, respectively.

Supplementary Table S3: **K-mer based genome size estimates (in bp) from unassembled RPW DNA-seq data.**

Method	This study 10x	Hazzouri et al.[1] 10x	Hazzouri *et al.*[1] ♀	Hazzouri *et al.*[1] ♂
findGSE	590,055,225	929,305,983†	592,913,934	569,174,629
GenomeScope	573,088,616	1,420,637,199†	577,505,135	554,924,223

† Genome size estimates based on the 10x Genomics library from Hazzouri *et al.*[1] (SRX5416727) are biased since this library was generated from multiple individuals and thus it does not fit the assumptions of k-mer based genome size estimation methods that assume diploidy.[2,3]

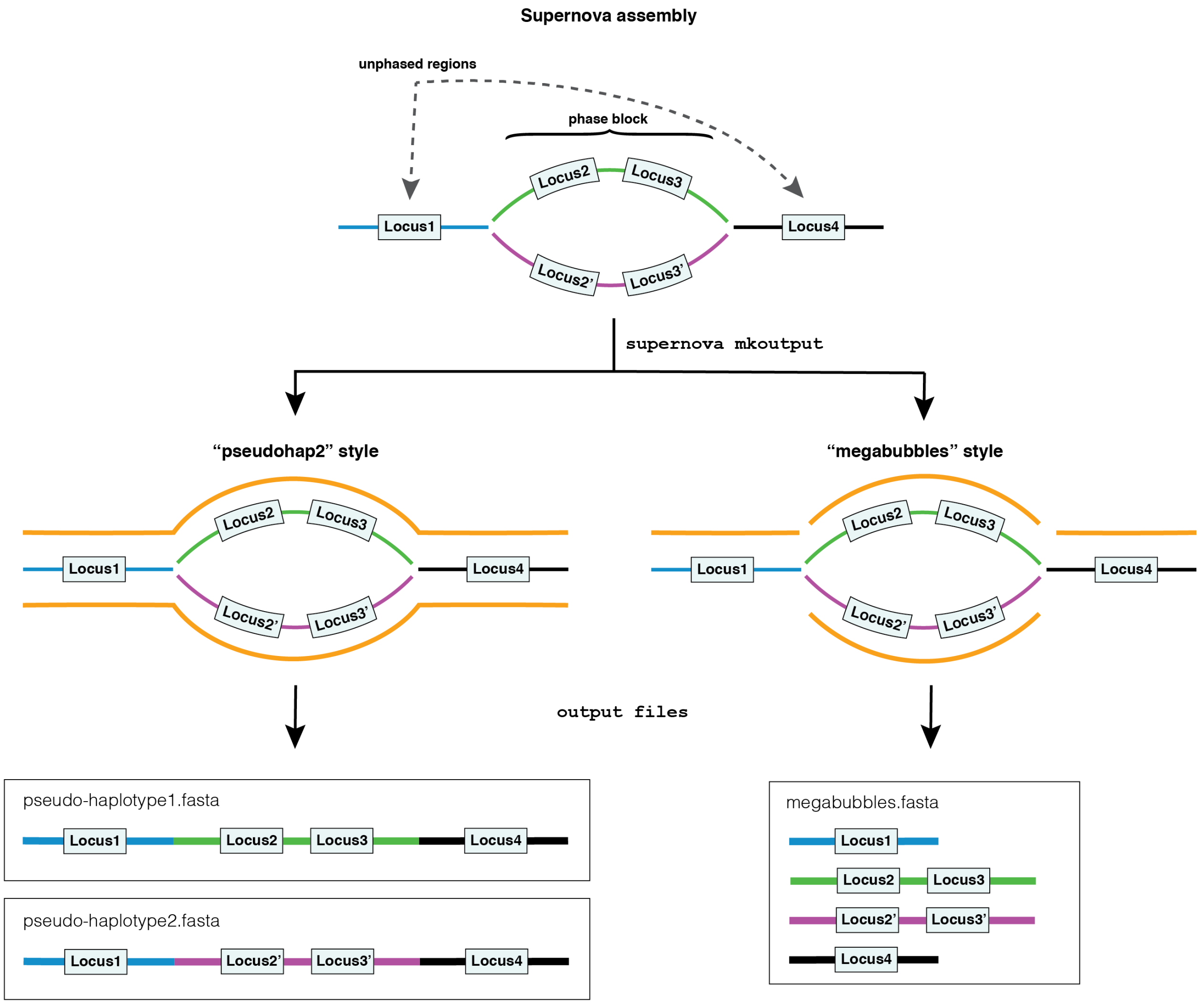

Supplementary Figure S1: **Overview of Supernova assembly output formats.** Supernova assemblies of 10x Genomics libraries can be exported in different output formats that represent phased and unphased regions of the genome in different styles.[4] The "pseudohap2" style generates two distinct "pseudo-haplotype" assembly files that differ in genomic regions where maternal and paternal haplotypes can be phased ("phase blocks") but are identical in homozygous and unphased blocks of the genome. Phasing occurs independently in each phase block and information about the linkage of maternal and paternal phase blocks does not span unphased regions. Thus, the resulting haploid assemblies contain a mixture of maternal and paternal segments and are considered pseudo-haplotypes rather than true haplotypes. Importantly, only one copy of each locus is included per pseudo-haplotype output file. The "megabubbles" style generates one assembly that includes maternal and paternal phase blocks together with unphased blocks in a single file. A megabubbles formatted Supernova assembly thus contains multiple haplotypes for the same locus in a single file, which can lead to haplotype-induced duplication artifacts when this format is used for scaffolding or analyzing gene content.

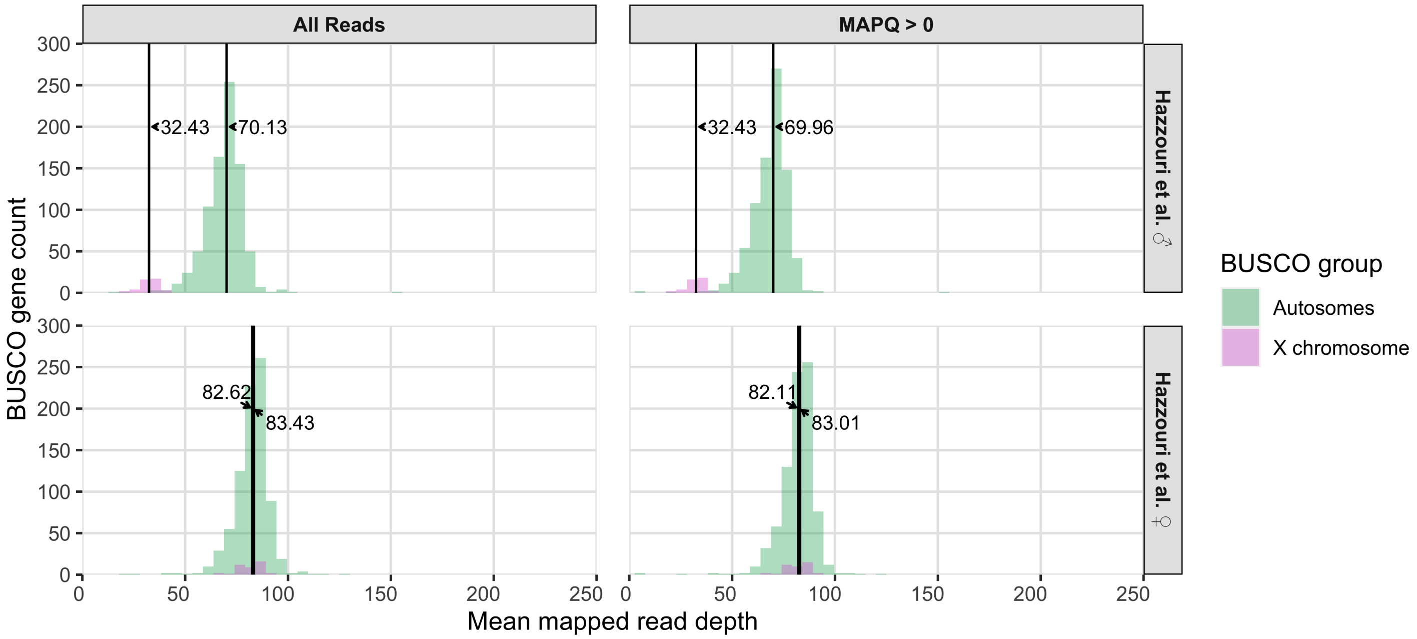

Supplementary Figure S2: **Mapped read depth of BUSCO genes in the RPW pseudo-haplotype1 assembly.** To test whether a depth-based approach could detect statistically significant changes in the copy number of BUSCO genes we calculated mean mapped read depth for each gene using male (SRX5416729) and female (SRX5416728) DNA sequencing data,[1] and then compared the depth distributions of genes on the X chromosome with those on autosomes using the Kolmogorov-Smirnov test. This approach correctly detects differences between X and autosomes in the male sample (reject null with $P \sim 0$), and not in the female sample (does not reject null, $P > 0.05$). The median of each distribution is indicated with a vertical line and depth estimates based on all mapped read and high quality mapped reads (MAPQ > 0) are shown in the left and right columns, respectively.

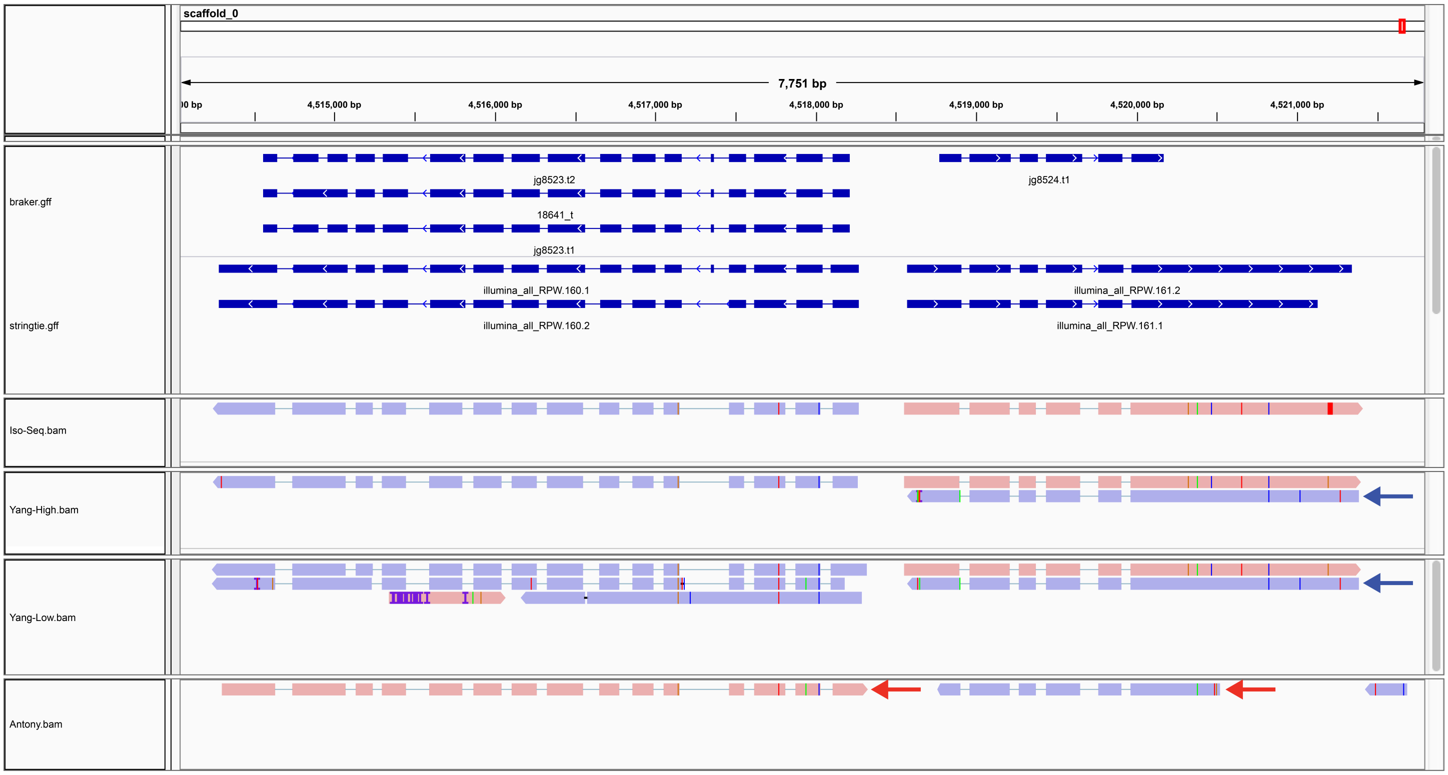

Supplementary Figure S3: **Integrated Genomics Viewer screenshot of strand orientation artifacts in published RPW transcriptomes.** Tracks from top to bottom are: transcript models from the BRAKER annotation of pseudo-haplotype1; StringTie transcript models based on short read RNA-seq; transcripts from our re-processing of long-read Iso-Seq data from Yang *et al.*;[5] transcripts from our the "high-quality" long-read Iso-Seq dataset from Yang *et al.*[5] (SRX7519788); transcripts from the "low-quality" long-read Iso-Seq dataset from Yang *et al.*[5] (SRX8694670); transcripts from the short-read transcriptome assembly from Antony *et al.*[6] (GDKA00000000). Transcript orientation is represented by arrowheads in the top two tracks and by color (red: forward; blue: reverse) and terminal exon direction in the bottom four tracks. Dark blue arrows highlight transcripts in the processed datasets from Yang *et al.*[5] that map to the opposite strand as BRAKER and StringTie models, and other Iso-Seq transcripts from the same locus. We interpret these to represent non-full length cDNA subreads that were sequenced on the anti-sense strand that were not filtered out in the Yang *et al.*[5] processed transcriptomes, but which are properly filtered in our isoseq3 re-processed version of the data from Yang *et al.*[5] Dark red arrows highlight transcripts in the transcriptome assembly from Antony *et al.*[6] that were assembled in the anti-sense orientation relative to BRAKER and StringTie models.

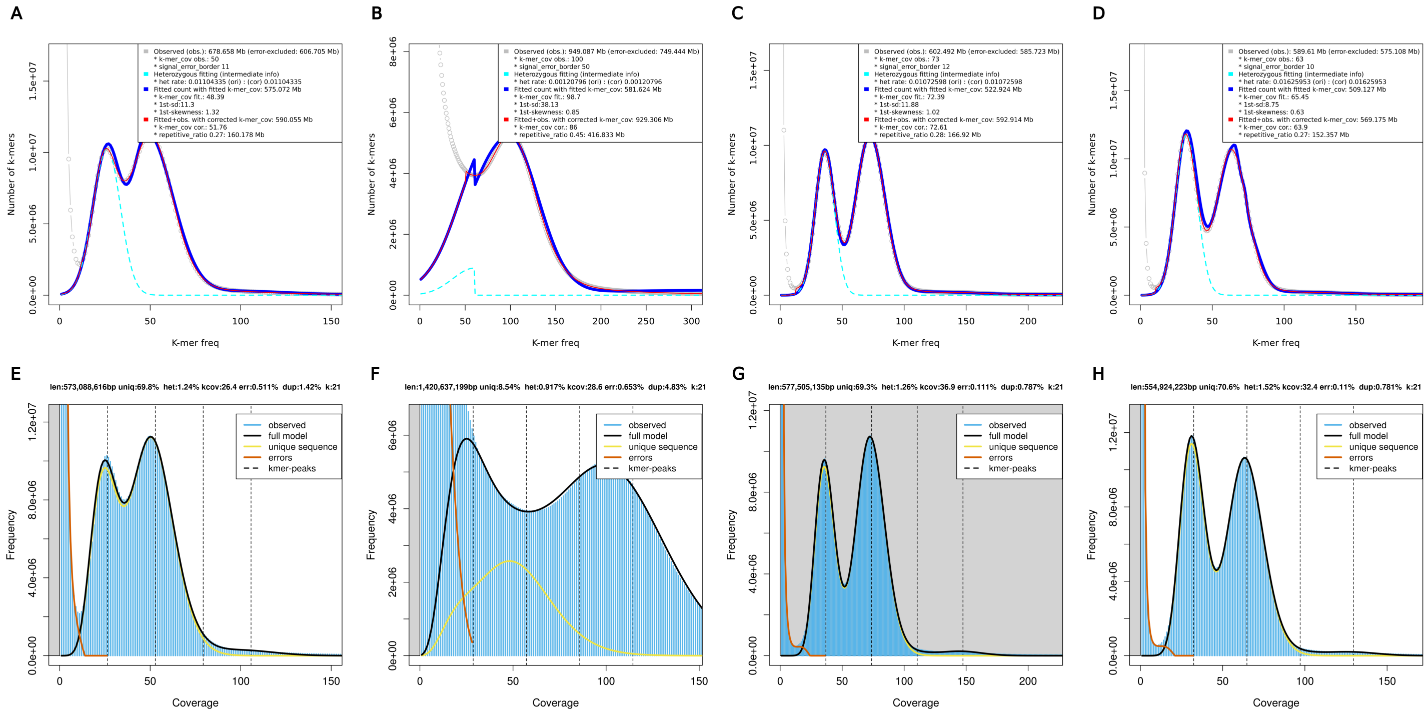

Supplementary Figure S4: **K-mer frequency profiles and genome size estimates for the RPW using unassembled DNA-seq reads.** Rows correspond to the two methods used for genome size estimation: (A-D) findGSE,[2] and (E-H) GenomeScope.[3] Columns correspond to the four RPW DNA-seq datasets analyzed and are arranged as follows: (A, E) 10x Genomics library from this study (SRX7520800); (B, F) 10x Genomics library from Hazzouri *et al.*[1] (SRX5416727); (C, G) female Illumina PE library from Hazzouri *et al.*[1] (SRX5416728); and (D, H) male Illumina PE library from Hazzouri *et al.*[1] (SRX5416729). All estimates were obtained from 21-mer frequency histograms with a max depth of 1,000,000. We note that estimates based on the 10x Genomics library from Hazzouri *et al.*[1] (SRX5416727) are biased since it was generated from multiple individuals and thus does not fit the assumptions of k-mer based genome size estimation methods that assume diploidy.[2,3]

References

[1] Khaled Michel Hazzouri, Naganeeswaran Sudalaimuthuasari, Biduth Kundu, David Nelson, Mohammad Ali Al-Deeb, Alain Le Mansour, Johnston J. Spencer, Claude Desplan, and Khaled M. A. Amiri. The genome of pest Rhynchophorus ferrugineus reveals gene families important at the plant-beetle interface. *Communications Biology*, 3(1):1–14, June 2020.

[2] Hequan Sun, Jia Ding, Mathieu Piednoël, and Korbinian Schneeberger. findGSE: estimating genome size variation within human and Arabidopsis using k-mer frequencies. *Bioinformatics*, 34(4):550–557, 2018.

[3] Gregory W. Vurture, Fritz J. Sedlazeck, Maria Nattestad, Charles J. Underwood, Han Fang, James Gurtowski, and Michael C. Schatz. GenomeScope: fast reference-free genome profiling from short reads. *Bioinformatics*, 33(14):2202–2204, July 2017.

[4] Neil I. Weisenfeld, Vijay Kumar, Preyas Shah, Deanna M. Church, and David B. Jaffe. Direct determination of diploid genome sequences. *Genome Res*, 27(5):757–767, 2017.

[5] Hongjun Yang, Danping Xu, Zhihang Zhuo, Jiameng Hu, and Baoqian Lu. SMRT sequencing of the full-length transcriptome of the Rhynchophorus ferrugineus (Coleoptera: Curculionidae). *PeerJ*, 8:e9133, May 2020.

[6] Binu Antony, Alan Soffan, Jernej Jakše, Mahmoud M. Abdelazim, Saleh A. Aldosari, Abdulrahman S. Aldawood, and Arnab Pain. Identification of the genes involved in odorant reception and detection in the palm weevil Rhynchophorus ferrugineus, an important quarantine pest, by antennal transcriptome analysis. *BMC Genomics*, 17(1):69, January 2016.
